# Stand, Einordnung und Potenzial der Implementierungswissenschaft: eine Bestandsaufnahme für Public Health in Deutschland

**DOI:** 10.1007/s00103-025-04077-7

**Published:** 2025-06-30

**Authors:** Heide Weishaar, Anna Kuehne, Kayvan Bozorgmehr, Hajo Zeeb

**Affiliations:** 1 Robert Koch-Institut, Gerichtstr. 27, 13347 Berlin, Deutschland; 2 Professur Öffentliche Gesundheit, Zentrum für Evidenzbasierte Gesundheitsversorgung (ZEGV), Universitätsklinikum und Medizinische Fakultät Carl Gustav Carus der TU Dresden, Dresden, Deutschland; 3 Fakultät für Gesundheitswissenschaften, Universität Bielefeld, AG Bevölkerungsmedizin und Versorgungsforschung, Bielefeld, Deutschland; 4 Leibniz-Institut für Präventionsforschung und Epidemiologie BIPS, Bremen, Deutschland; 5 Health Sciences Bremen, Universität Bremen, Bremen, Deutschland

**Keywords:** Implementierungsforschung, Theorien, Modelle und Frameworks (TMFs), Versorgungsforschung, Equity, Evidenz, Implementation research, Implementation science theories, models, and frameworks (TMFs), Germany, Health care research, Evidence

## Abstract

Die Implementierung von qualitativ hochwertigen, evidenzbasierten Maßnahmen zur Verbesserung der Bevölkerungsgesundheit ist eine Voraussetzung und ein Kernelement von Public Health. Oft bestehen jedoch Lücken zwischen Forschung und Praxis, was zu einer Unterversorgung mit wirksamen Interventionen, zu Fehlversorgung mit ungeeigneten Interventionen und geringwertiger Versorgung führt. Die Implementierungswissenschaft zielt darauf, eine Brücke zwischen Wissensproduktion und Umsetzung zu schlagen, indem sie Evidenz zur Integration von Forschungsbefunden und evidenzbasierten Praktiken in Handlungsroutinen generiert. Dieser Diskussionsbeitrag gibt einen Einblick in wichtige Aspekte der Implementierungswissenschaft und beschreibt Entwicklungen in Deutschland. Zunächst erfolgen eine Einordnung der Implementierungswissenschaft sowie eine Darstellung ausgewählter Theorien, Modelle und Frameworks, die in der Implementierungsforschung angewandt werden, um Implementierungsstrategien, Einflussfaktoren und Outcomes von Interventionen systematisch zu analysieren. Anschließend wird die Implementierungswissenschaft in Deutschland im Hinblick auf konkrete Projekte, die institutionelle Verankerung und Anbindung, die Lehre und die Vernetzung beleuchtet. Der Artikel schließt mit einer kritischen Reflexion bezüglich der Grenzen und Potenziale der Implementierungswissenschaft für Public Health in Deutschland.

Eine englische Volltextversion dieses Artikels ist bei SpringerLink als Onlinematerial verfügbar.

**Zusatzmaterial online:**

Zusätzliche Informationen sind in der Online-Version dieses Artikels (10.1007/s00103-025-04077-7) enthalten.

## Abstract (en)

The implementation of high-quality, evidence-based practice to improve the health of populations is a prerequisite and a core element of public health. However, gaps frequently exist between research and practice, leading to the underuse of effective interventions and misuse of inappropriate interventions. Implementation science aims to bridge the gap between knowledge production and implementation by generating evidence to improve the transfer of research findings and evidence-based practices into routine action. This discussion article reflects on the state of implementation science in Germany. First, we provide a descriptive classification of implementation science, followed by a review of selected theories, models, and frameworks that are used in implementation research to systematically analyze implementation strategies, barriers, facilitators, and outcomes of interventions. Subsequently, implementation science in Germany is examined with regard to specific projects, institutional integration, teaching, and networking among experts. The article concludes with a critical reflection on the limitations and potential of implementation science for public health in Germany.

An English full-text version of this article is available at SpringerLink as Supplementary Information.

## Was ist Implementierungswissenschaft?

Die Implementierungswissenschaft etabliert sich seit einigen Jahren international und auch national als eine eigenständige Forschungsrichtung, die in engem Bezug insbesondere zur Versorgungsforschung steht. Das Ziel des vorliegenden Diskussionsbeitrags ist es, Fragestellungen und Konzepte der Implementierungswissenschaft aufzuzeigen und kritisch einzuordnen, den Stand der Implementierungswissenschaft in Deutschland – ohne Anspruch auf Vollständigkeit – zu skizzieren und zukünftige Ausrichtungen und Entwicklungspotenziale zu diskutieren.

Medizinische, epidemiologische und Public-Health-Forschung liefert Erkenntnisse dazu, wie bestimmte individuelle, organisationsbezogene oder strukturelle Einflussfaktoren verändert oder Maßnahmen gestaltet werden sollten, um die gesundheitliche Versorgung bzw. die Gesundheit der Bevölkerung zu verbessern. Dabei liegt der Fokus oftmals auf der Wirksamkeit unter spezifischen Bedingungen und auf bestimmten vorselektierten Personengruppen. Deutlich weniger ist bekannt über die Umsetzung von Maßnahmen und Programmen unter Alltagsbedingungen, welche sich i. d. R. deutlich von der Umsetzung in stärker kontrollierten Forschungssettings unterscheidet. Dies führt häufig zu geringer externer Validität und Implementierung.

Die limitierte Integration von Evidenz drückt sich unter anderem in langen Implementierungszeiten aus: Die Implementierung von Maßnahmen im medizinischen Kontext dauert im Durchschnitt 17 Jahre [1] und im Public-Health-Kontext kann die Implementierung von populationsbezogenen Maßnahmen noch länger dauern, wie die mehrere Dekaden andauernde Entwicklung und die teils lückenhafte Umsetzung effektiver Tabakkontrollprogramme zeigen [2, 3]. Darüber hinaus kommt es aufgrund fehlender Evaluierung und wissenschaftlicher Begleitung von Maßnahmen nur selten bzw. verzögert zur Deimplementierung von Maßnahmen, die nicht die erwünschte Wirkungen zeigen [4]. Die fehlende oder verzögerte Implementierung wirksamer sowie Deimplementierung unwirksamer Maßnahmen tragen zur Unterversorgung mit innovativen, nachweislich wirksamen Interventionen und zur Fehl- und Überversorgung mit unwirksamen Interventionen bei; qualitativ hochwertige evidenzbasierte Interventionen werden nicht oder nicht wirksam eingesetzt [5]. Hier setzt die Implementierungsforschung an, um mittels multi- und interdisziplinärer Forschung unter Verwendung von beispielsweise qualitativen und quantitativen Methoden aus den Sozial- und Gesundheitswissenschaften, der Epidemiologie sowie aus anderen wissenschaftlichen Disziplinen die Lücke zwischen Wissensproduktion und Umsetzung zu verkleinern.

Die Implementierungswissenschaft umfasst die Implementierungsforschung im Bereich der gesundheitlichen Versorgung, Bevölkerungsgesundheit und Gesundheitspolitik [6]. Die Definition der Implementierungsforschung beinhaltet folgende Aspekte:

- Implementierungsforschung beschäftigt sich mit Implementierungsstrategien zur Förderung der systematischen Integration von Forschungsbefunden und evidenzbasierten Praktiken in Handlungsroutinen; sie trägt somit zur Verbesserung der Qualität und Effektivität von Gesundheitsdiensten bei [1, 6, 7].
- Implementierungsforschung ist auf die komplexen Fragestellungen und Herausforderungen in der *praktischen* Umsetzung von Innovationen ausgerichtet und bezieht dabei auch regelmäßig Fragen der Nachhaltigkeit und des Kapazitätenaufbaus ein [8, 9].
- Implementierungsforschung interessiert sich für *umsetzungsbezogene* Ergebnisparameter wie Akzeptabilität, Umsetzungsbereitschaft, Machbarkeit, Angemessenheit, Kosten, Umsetzungstreue, Durchdringung und Nachhaltigkeit [9].

Evidenzbasierung im Sinne einer grundsätzlichen Wirksamkeit ist daher Voraussetzung und nicht Kernthema dieser Forschung. Es geht darum, konkrete, umfassende Erkenntnisse zur Umsetzung gesundheitsbezogener Interventionen unter den oft stark variierenden Bedingungen in unterschiedlichen Kontexten und für diverse Zielgruppen zu gewinnen. Dies schließt auch Forschung zur *De*implementierung, also zur Beendigung von in der Praxis unwirksamen oder sogar mit negativen Folgen verbundenen Maßnahmen ein, die Teil etablierter Handlungsroutinen sind [10].

## Wie komplementiert die Implementierungswissenschaft andere Forschungsgebiete?

**Abb. 1:**
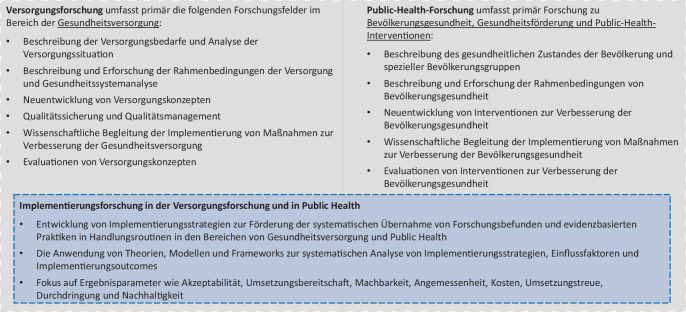
Schematische Darstellung von thematischen Schwerpunkten und Bezügen von Implementierungsforschung im Bereich Public-Health- und Versorgungsforschung

Die Implementierungsforschung wird in vielfältigen Forschungsbereichen angewandt, um die komplexen Wechselwirkungen zwischen Interventionen und deren Umwelt systematisch zu analysieren. Entsprechende Analysen erfolgen auch in der Versorgungs- und Public-Health-Forschung ([8, 11–13]; Abb. 1). Eine Stellungnahme der Deutschen Forschungsgemeinschaft (DFG) von 2008 beschreibt die Versorgungsforschung als Disziplin, deren Fokus eher auf Erkrankten oder Erkrankungsgefährdeten und medizinischen Maßnahmen liegt, während Public Health sich vorrangig mit der Gesunderhaltung sowie nichtmedizinischen Maßnahmen beschäftigt [14]. Dieser Logik folgend umfasst die Implementierungsforschung im Public-Health-Bereich die Erforschung von Implementierungsstrategien, Einflussfaktoren und Implementierungsoutcomes in Bezug auf komplexe Public-Health-Maßnahmen mit dem Ziel der Gesundheitsförderung [15]. Implementierungsforschung in der Versorgungsforschung konzentriert sich hingegen auf Interventionen mit einem individualmedizinischen Fokus von Früherkennung, kurativer medizinischer Versorgung und Rehabilitation [16, 17]. Die Implementierungsforschung umfasst jedoch nicht die Forschungsfelder der Bedarfserhebung, Beschreibung des Versorgungszustandes oder der grundsätzlichen Wirksamkeitsevaluation, die zur Versorgungsforschung gehören [16–18], und auch nicht andere charakteristische Public-Health-Forschungsfelder. Zudem erhält in der Implementierungsforschung die Partizipation der für die jeweilige Maßnahme relevanten Akteursgruppen an der Forschungsgestaltung eine große Gewichtung [19–21]. Weiterhin liegt ein Fokus mehrerer Modelle und Frameworks der Implementierungsforschung stärker auf dem Aspekt der Akzeptabilität (bei Durchführenden und Nutzenden) und der Passung der Intervention sowie der Nachhaltigkeit der Implementierung [17, 18].

## Theorien, Kernkonzepte, Modelle und Frameworks der Implementierungswissenschaft

Primär fokussiert die Implementierungswissenschaft darauf, Erkenntnisse zu gewinnen, die die Integration von Forschungsergebnissen in die Praxis wahrscheinlicher bzw. effektiver machen bzw. die es ermöglichen, Interventionen zu verbessern und anzupassen und so die Kluft zwischen Wissen und Handeln („know-do-gap“) zu verringern. Sie beschäftigt sich umfassend mit der Frage, welche Einflussfaktoren fördernd oder hemmend auf die Umsetzung von Maßnahmen wirken und welche Implementierungsstrategien für die Umsetzung förderlich sein können. Entsprechend gibt es Ergebnisparameter, die üblicherweise in der Implementierungswissenschaft untersucht werden und die als Implementierungsoutcomes bezeichnet werden.

Als Indikatoren für den Erfolg der Implementierung fungieren Implementierungsoutcomes als Mediatoren zwischen einer Intervention und ihrem Erfolg, denn eine Intervention kann nur erfolgreich sein, wenn sie adäquat implementiert wird [9]. In der Implementierungswissenschaft wird entsprechend zwischen der Effektivität der *Implementierung* und der Effektivität der *Intervention* unterschieden. Wenn die Umsetzung von Forschungsergebnissen nicht funktioniert, weil die Intervention unpassend ist, spricht man von einem Interventionsfehler; wenn die Umsetzung scheitert, weil eine passende Intervention falsch umgesetzt wurde, spricht man von einem Implementierungsfehler.

In der Implementierungsforschung wird zudem ein Schwerpunkt auf das Einhalten der Vorgaben einer Intervention im Hinblick auf die Genauigkeit der Umsetzung oder Umsetzungstreue (englisch: „fidelity“) gelegt [9]. Hierbei wird auf Implementierungsquantität (d. h. die Vollständigkeit der Intervention) und Implementierungsqualität (d. h. die Genauigkeit in der Umsetzung der Intervention) geachtet. Es können jedoch Änderungen bei der Implementierung von Interventionen nötig sein, um die Intervention an das spezifische Setting anzupassen.

Um Einflussfaktoren zu identifizieren und Ergebnisparameter zu bewerten, sind deren systematische Erfassung und Analyse hilfreich. In der Implementierungswissenschaft werden Theorien, Modelle und Frameworks (englisch: „theories, models, frameworks“ – TMFs) angewandt, um systematische Analysen zu unterstützen. TMFs helfen dabei, Implementierungsstrategien auszuwählen oder zu entwickeln, Einflussfaktoren auf die Implementierung zu beschreiben und Outcomes zu messen. Mittlerweile existiert eine Vielzahl von TMFs, die teilweise Überschneidungen und Synergien aufweisen [22]. Eine Übersichtsarbeit von Tabak et al. zur Disseminations- und Implementierungsforschung aus dem Jahr 2012 identifiziert insgesamt 61 TMFs [22]. Die Übersicht ist praktisch ausgerichtet und will Forschenden erleichtern, passende Konstrukte für ihre eigene Forschung anhand zentraler Kategorien (Fokus auf Dissemination oder Implementierung, Konstruktflexibilität, sozioökologischer Level) auszuwählen. Ein spätere Übersichtsarbeit von Nilsen [23] schlägt eine Taxonomie von TMFs vor, die über die Ordnungsprinzipien von Tabak et al. [22] hinausgeht. Nilsens Taxonomie zufolge dienen TMFs in der Implementierungswissenschaft 3 wesentlichen Zielen:

1. der Beschreibung und/oder Anleitung des Integrationsprozesses von der Forschung in die Praxis,
2. dem Verständnis/der Erklärung von Einflussfaktoren auf die Implementierungsoutcomes und
3. der Evaluation von Implementierung.

Diesen 3 Zielen ordnet Nilsen 5 verschiedene TMF-Kategorien zu, so etwa die Prozessmodelle dem ersten Ziel bzw. die Determinantenmodelle und die klassischen Theorien dem zweiten Ziel. Exemplarisch werden hier kurz 2 etablierte Frameworks erläutert, die sich stark auf den Kontext beziehen, in dem gesundheitsbezogene Innovationen umgesetzt werden.

Das „Consolidated Framework for Implementation Research“ (CFIR; [11, 13]) bietet einen umfassenden und auf klassische Theorien aus der Soziologie und Psychologie zurückgreifenden Ansatz, um Umgebungsaspekte von Interventionen – sogenannte kontextuelle Determinanten – systematisch zu erfassen. Die identifizierten Förderfaktoren und Barrieren können dann in der Ausrichtung und Anpassung von Implementierungsstrategien berücksichtigt werden. Das mittlerweile in die deutsche Sprache übertragene CFIR weist 5 grundsätzliche Domänen auf [24], die jeweils mit Detailkriterien und genauen Beschreibungen erläutert werden und so die Anwendung des Frameworks erleichtern. Die Domänen sind: Charakteristika der Intervention, äußeres Setting, inneres Setting, Charakteristika der Individuen sowie Prozesse. Einige dieser Domänen wurden in einer Überarbeitung des Frameworks [13] angepasst und erweitert, z. B. wurden Outcomes aufgenommen. Die Autoren heben hervor, dass nicht alle Domänen für jede Intervention und Implementierung von gleicher Bedeutung sind und dass das CFIR flexibel genutzt werden soll.

Das Framework „Context and Implementation of Complex Interventions“ (CICI) nimmt die Implementierung und den Kontext komplexer Interventionen in den Fokus [12]. Es begründet dies damit, dass für komplexe Interventionen die umgebenden Rahmenbedingungen von größter Bedeutung sind. Dabei greift das CICI-Framework systematisch auf Vorgängermodelle, einschließlich des CFIR, zurück und analysiert diese in Hinsicht auf die Besonderheiten von komplexen Interventionen. Im CICI-Framework werden für die 3 Dimensionen Kontext, Implementierung und Setting unterschiedliche Domänen spezifiziert, die eine differenzierte Zuordnung spezifischer Interventionskomponenten ermöglichen und so die Interaktion zwischen verschiedenen Komponenten und dem jeweiligen Setting transparent machen. Das CICI-Framework regt mittels einer Checkliste an, systematisch über geografische, epidemiologische, soziokulturelle, ökonomische, ethische, rechtliche und politische Kontexte in Bezug auf die jeweilige Intervention zu reflektieren. Pfadenhauer et al. demonstrieren die Anwendung und den Erkenntnisgewinn aus dem Framework anhand eines konkreten Beispiels, der Implementierung von Maßnahmen zur Verbesserung der Luftqualität in Irland [12].

Um den Erfolg von Implementierungsprozessen zu messen, fokussiert Proctors Taxonomie [25] spezifisch auf die Bewertung von Implementierungsoutcomes. Sie hebt verschiedene Dimensionen hervor, die wichtig sind, um die Annahme und Nutzung von Interventionen in der Praxis zu bewerten. Dabei werden die Domänen Akzeptabilität, Umsetzungsbereitschaft, Machbarkeit, Angemessenheit, Kosten, Umsetzungstreue, Durchdringung und Nachhaltigkeit betrachtet [25].

In der Abwägung der Nutzung adäquater Frameworks gilt es, deren Ausrichtung mit dem eigenen Forschungsgegenstand kritisch zu prüfen [22]. CFIR zielt auf die Identifikation von Einflussfaktoren ab und hat einen starken Kontextfokus: Es bietet eine pragmatische Struktur zum genaueren Verständnis von Implementationsprozessen und -ergebnissen und nimmt die individuellen Akteure (z. B. deren Motivation und Kompetenzen) in den Blick [11]. Wenn es um komplexe Public-Health-Interventionen und die Interaktion der verschiedenen Aspekte mit den Settings geht, kann CICI geeignet sein, insbesondere wenn settingübergreifend gearbeitet wird [12]. In Hinblick auf eine genaue Operationalisierung von Ergebnisparametern kann Proctors Taxonomie der Implementierungsoutcomes zu Rate gezogen werden: Sie verwendet klare Metriken, die qualitativ oder quantitativ operationalisiert und auf verschiedene Sektoren (Gesundheitswesen, Bildung, Sozialdienste) angewandt werden können [25].

## Wie ist Implementierungswissenschaft in Deutschland vertreten?

Implementierungswissenschaft findet man in vielen Bereichen der Public-Health- und Versorgungsforschung. Im Folgenden skizzieren wir den Stand der Implementierungswissenschaft in Deutschland, ohne Anspruch auf Vollständigkeit und unter Anerkennung der Tatsache, dass die o. g. Elemente und Aspekte der Implementierungswissenschaft über die im Folgenden genannten Bereiche hinaus in der deutschen Forschungslandschaft vertreten sein können. Zuerst stellen wir 4 Beispiele aus dem deutschsprachigen Kontext vor, um die Vielfältigkeit und Bandbreite der Implementierungsforschung im Gesundheitsbereich zu veranschaulichen.

### Sport- und Bewegungstherapie bei Krebspatient*innen.

In 2 großen Verbundforschungsprojekten, die von der Deutschen Krebshilfe gefördert werden, untersuchen interdisziplinäre Forschungsteams standortübergreifend, wie eine qualitätsgesicherte Sport- und Bewegungstherapie bei Krebspatient*innen in Deutschland breiter implementiert werden kann. Die Effektivität der Sport- und Bewegungstherapie ist seit vielen Jahren in Bezug auf die Verbesserung von Lebensqualität und eine Reihe weiterer Outcomes nachgewiesen [26], wird aber bisher unzureichend in der onkologischen Versorgungsroutine eingesetzt. Die Projekte IMPLEMENT^1^ und MOVE-ONKO^2^ untersuchen nun auf der Basis implementierungswissenschaftlicher Methoden, welche Barrieren und Förderfaktoren unterschiedliche Implementierungsstrategien beeinflussen und wie diese in konkreten Pilotprojekten berücksichtigt werden können, um später die bundesweite Implementierung der qualitätsgesicherten Sport- und Bewegungstherapie bei Krebspatient*innen zu befördern.

### Tabakkontrolle.

Die Forschung zu Maßnahmen zur Eindämmung des Tabakkonsums und insbesondere das International Tobacco Control (ITC) Policy Evaluation Project^3^ stellen Beispiele für Implementierungsforschung dar. Das ITC-Projekt wird von Wissenschaftler*innen der University of Waterloo in Kanada geleitet; das Deutsche Krebsforschungszentrum ist als deutscher Kooperationspartner beteiligt. Ziel der internationalen Kohortenstudie zum Tabakkonsum ist die systematische und umfassende Evaluation verschiedener Maßnahmen der Tabakprävention und Tabakkontrolle^8^. Dies beinhaltet die Evaluation von politischen Interventionen (z. B. von Gesetzen zum Passivrauchschutz, Preis- und Steuerpolitik, gesetzlich regulierten Warnhinweisen) und die Identifikation von den Faktoren, die auf eine effektive Tabakkontrollpolitik Einfluss haben^8^.

### Begleitforschung des Nationalen Zentrums für Frühe Hilfen (NZFH).

Die begleitende Forschung des NZFH^4^ kann als ein Beispiel für Implementierungsforschung in Public Health in Deutschland gewertet werden. So erhebt das NZFH regelmäßig im Rahmen einer Begleitevaluation Daten zum strukturellen Aus- und Aufbau der Frühen Hilfen und analysiert deren Umsetzung, z. B. in Bezug auf formale Zuständigkeiten, die Verankerung der Frühen Hilfen in der kommunalen Planung und Steuerung sowie Ausstattung und Qualitätsmerkmale der Frühen Hilfen^12^. Die Implementierung der Frühen Hilfen im Umsetzungsalltag wird anhand von konkreten Indikatoren evaluiert^12^.

### Implementierungsforschung im Öffentlichen Gesundheitsdienst.

Implementierungsforschung kann einen wesentlichen Beitrag zur erfolgreichen Implementierung neuer evidenzbasierter Maßnahmen im Öffentlichen Gesundheitsdienst (ÖGD) leisten. Während die Anzahl wissenschaftlich publizierter projektbezogener Evaluationen und Projekte der angewandten Forschung im Bereich der Gesundheitsförderung, -vorsorge und -versorgung im ÖGD in den vergangenen 20 Jahren deutlich gestiegen ist, sind systematische Ansätze der Implementierungsforschung noch selten [27–30]. Im Bereich der strukturierten Intra- und After-Action-Reviews als Methode der Anpassung und nachhaltigen Verbesserung des Krisenmanagements im ÖGD nach einer Krise und in Vorbereitung auf zukünftige Krisen [31, 32] zeigen sich erste systematische Ansätze, die in Hinblick auf die strukturierte Erfassung von Gelingensfaktoren Ähnlichkeiten zur Implementierungsforschung aufweisen [28, 33, 34].

Eine Studie von Schultes et al. [35] identifiziert die folgenden 7 Barrieren und fördernden Faktoren für die Durchführung von Implementierungswissenschaft in deutschsprachigen Ländern:

i. die Merkmale der Implementierungswissenschaft als wissenschaftliche Disziplin, einschließlich eines Mangels eines gemeinsamen Verständnisses dafür, was als Implementierungswissenschaft bezeichnet werden kann;
ii. die Bedingungen und besonderen Herausforderungen für entsprechende Forschungsprojekte;
iii. persönliche, mit der Durchführung zusammenhängende Einflussfaktoren;
iv. die (geringe) Vernetzung zwischen Implementierungswissenschaftler*innen;
v. die (geringen) Möglichkeiten zum Kompetenzerwerb;
vi. die (unzureichende) finanzielle Förderung von Implementierungsforschung und
vii. die (geringe) Bereitschaft, Implementierungswissenschaft im wissenschaftlichen Kontext zu etablieren.

Entsprechend dem letzten Punkt ist die Implementierungswissenschaft in Deutschland im Vergleich zum angloamerikanischen Raum bislang nicht breit institutionell verankert [35]. Nur wenige wissenschaftliche Institutionen schenken dem Thema explizit Aufmerksamkeit. Eine designierte Professur für Versorgungsforschung und Implementierungswissenschaft gibt es nur an der Universität Heidelberg^5^. An der Universität Bremen gibt es 2 Professuren, die sich mit Implementierungswissenschaft in 2 Spezialgebieten von Gesundheit befassen,^6^ und Anfang 2025 wurde an der Universität Leipzig eine Stiftungsprofessur mit Bezug zu Implementierungswissenschaft eingerichtet^7^. Darüber hinaus gibt es einzelne wissenschaftliche Einrichtungen, welche die Implementierungswissenschaft im Rahmen von Forschungs- oder Arbeitsgruppen in den Fokus stellen. Besonders häufig ist die Implementierungswissenschaft an Fachhochschulen angesiedelt, was vermutlich mit der Anwendungsnähe im Zusammenhang steht.

Einen Überblick über Fortbildungen und Kurse in der Implementierungswissenschaft in Europa bietet eine europaweite Datenbank der European Implementation Collaborative^8^. Die meisten Fortbildungen und Kurse werden im Rahmen von Studiengängen angeboten und sind Teil von Master- und Promotionsstudiengängen; jedoch gibt es auch etliche Kurse zur beruflichen Weiterbildung^9^. In Heidelberg ist der Versorgungsforschung und Implementierungswissenschaft im Gesundheitswesen ein Masterstudiengang gewidmet.^9^ An der Fliedner Fachhochschule gibt es einen berufsbegleitenden Zertifikatskurs zum*r Implementierungsmanager*in Healthcare^10^. Darüber hinaus zeigt eine pragmatische Online-Suche, dass ein Teil der Lehre in der Implementierungswissenschaft in Deutschland an Fachhochschulen bzw. innerhalb von Studiengängen für Gesundheitsfachberufe angesiedelt ist.^11^

Wie der oben dargestellte Überblick zeigt, steht eine breite Verankerung der Implementierungswissenschaft in der akademischen Lehre und Forschungslandschaft in Deutschland noch aus [35]. Hürden bestehen unter anderem darin, dass die Terminologie, die sich hauptsächlich im anglophonen Raum etabliert hat, im deutschsprachigen Raum nur bedingt anschlussfähig ist [35]. Die finanzielle Förderung für Implementierungsforschung findet zumeist im Rahmen übergreifender Förderprogramme wie etwa dem Innovationsfonds des Gemeinsamen Bundesausschusses (G-BA) der Krankenkassen statt und dieser fokussiert hauptsächlich auf Versorgungsforschungsprojekte im engeren Sinne. Andere Förderformate, wie die der DFG, bieten aufgrund ihrer Fokussierung auf Grundlagenforschung bisher wenig Anschlussfähigkeit an die Fragestellungen und Methoden sowie den Realweltbezug der Implementierungsforschung. Bestehende Förderprogramme heben zwar vermehrt die Notwendigkeit der Integration von Forschung in die Praxis hervor, eine explizite Finanzierung von Forschungsprojekten, die Einflussfaktoren und Strategien der Umsetzung untersuchen, ist jedoch nur eingeschränkt vorhanden.

Die Vernetzung und der Austausch von Wissenschaftler*innen ist auch für den Forschungsbereich Implementierungswissenschaft von großer Bedeutung. Nationale und internationale Netzwerke sowie wissenschaftliche Zeitschriften konzentrieren sich auf die Implementierungswissenschaft. Neben mehreren internationalen Zeitschriften, die den Fokus auf Implementierungswissenschaft legen, hat in Deutschland im September 2023 das Deutsche Netzwerk Versorgungsforschung (DNVF) erstmals das Journal *Health Care Research & Implementation* als Supplement des Journals *Das Gesundheitswesen* herausgegeben.^12^ Implementierungswissenschaftliche Themen sind natürlich nicht nur in spezifischen Zeitschriften zu finden, sondern auch in einer Vielzahl von Fachzeitschriften aus medizinischen, Public-Health- und anderen Bereichen. Eine internationale Analyse von Publikationen aus der Implementierungswissenschaft zeigt, dass die Identifikation von relevanter Literatur aufgrund der unspezifischen und variierenden Terminologie, die über mehrere Fachgebiete verteilt ist, schwierig ist [36]. In Tab. 1 sind – ohne Anspruch auf Vollständigkeit – relevante Netzwerke und Publikationsorgane im Bereich der Implementierungswissenschaft aufgelistet.

**Tab. 1:** Relevante Netzwerke und Publikationsorgane mit Bezug zur Implementierungswissenschaft

Titel	Beschreibung	Webseite
*Netzwerke*		
Deutsches Netzwerk Versorgungsforschung e. V. (DNVF), Arbeitsgruppe Implementierungswissenschaft und -praxis in der Versorgungsforschung	Netzwerk für Wissenschaftler*innen, die mit der Verbesserung der Gesundheits- und Krankenversorgung unter wissenschaftlichen, praktischen oder gesundheitspolitischen Gesichtspunkten befasst sind; seit 2024 gibt es eine Arbeitsgruppe Implementierungswissenschaft und -praxis in der Versorgungsforschung	https://www.dnvf.de/%C3%BCber-uns/%C3%BCber-das-netzwerk.html
Implementierungs-Netzwerk für Forschung und Praxis (INFo-P)	Implementierungsnetzwerk für Forschung und Praxis zur Vernetzung im deutschsprachigen Raum (inklusive Schweiz und Österreich)	https://www.implementierung.eu/
German Alliance for Global Health Research (GLOHRA)	Fokus auf globaler Gesundheit, bringt in verschiedenen Formaten an Implementierungswissenschaft interessierte Wissenschaftler*innen zusammen und bietet auch finanzielle Forschungsförderung in diesem Rahmen	https://globalhealth.de/about.html
European Implementation Collaborative (EIC)	Europäisches Forum für Wissenschaftler*innen, Praktiker*innen und Entscheidungsträger*innen, die sich für die Forschung und Praxis der Implementierung von Interventionen im Gesundheitsbereich interessieren	https://www.uni-heidelberg.de/de/studium/alle-studienfaecher/versorgungsforschung-und-implementierungswissenschaft-im-gesundheitswesen/versorgungsforschung-und-implementierungswissenschaft-im-gesundheitswesen-master
Global Implementation Society (GIS)	Netzwerk mit Fokus auf der Förderung und Etablierung von kohärenten und kooperativen Ansätzen für die Umsetzungspraxis, Wissenschaft und Politik	https://globalimplementation.org/
Society for Implementation Research Collaboration (SIRC)	Internationale wissenschaftliche Gesellschaft, die sich der Förderung der Kommunikation und Zusammenarbeit zwischen Forschenden und Interessengruppen widmet, die sich für die Bewertung der Umsetzung evidenzbasierter Maßnahmen einsetzen	https://societyforimplementationresearchcollaboration.org/
*Publikationsorgane*		
Implementation Science	Führendes internationales Journal in Implementierungswissenschaft, Schwerpunkt auf der Forschung zur Implementierung von Interventionen im Gesundheitswesen	https://implementationscience.biomedcentral.com/
Implementation Science Communications	Führendes internationales Journal in Implementierungswissenschaft, Schwerpunkt auf der Forschung zur Implementierung von Interventionen im Gesundheitswesen	https://implementationsciencecomms.biomedcentral.com/
Journal Global Implementation Research and Applications	Offizielles Journal der Global Implementation Society, Forum für die Entwicklung, Integration und den Austausch von Wissen und Erfahrungen im Bereich der Implementierung von Maßnahmen in verschiedensten Bereichen	https://link.springer.com/journal/43477
Journal of Implementation Science	Internationales Journal, Fokus auf Förderung der Praxis der Implementierungswissenschaft	https://openaccesspub.org/journal/implementation-science
Frontiers in Health Services, Section Implementation Science	Internationales Journal, Fokus auf Artikel, die die Weiterentwicklung der Implementierungswissenschaft im Gesundheits- und Sozialwesen vorantreiben	https://www.frontiersin.org/journals/health-services/sections/implementation-science
Implementation Research and Practice	Internationales Journal zu interdisziplinärer Forschung zur Umsetzung wirksamer Ansätze, Fokus auf psychische Erkrankungen und Sucht, mit Schwerpunkt auf vulnerablen Gruppen	https://us.sagepub.com/en-us/nam/implementation-research-and-practice/journal203691
Health Care Research & Implementation (als Supplement der Zeitschrift „Das Gesundheitswesen“)	Deutschsprachiges Journal, Fokus auf Studien mit Praxisbezug aus der Versorgungs- und Implementierungsforschung	https://dnvf.de/ver%C3%B6ffentlichungen/dnvf-journal.html
Health Policy & Planning	Journal für Gesundheitspolitik und Gesundheitssystemforschung mit Schwerpunkt auf Ländern mit niedrigem und mittlerem Einkommen, befasst sich mit Fragen, die für politische Entscheidungsträger*innen, Forschende und Praktiker*innen relevant sind, Fokus auf gesundheitspolitische und populationsbezogene Maßnahmen	https://academic.oup.com/heapol

## Kritik an der Implementierungswissenschaft

Trotz starker Stimmen, die die Vorteile der Implementierungswissenschaft hervorheben, ist der Forschungsbereich auch mit Kritik konfrontiert. Ein Kritikpunkt lautet, dass die schiere Anzahl an TMFs, Begrifflichkeiten und Ansätzen zu einer Fragmentierung sowie Silobildung des Felds geführt hat [37], welche integrative Erkenntnisse verhindert. Zudem werden mangelnde oder ausbaufähige Praxisrelevanz der Forschung [38, 39] sowie Entkopplung der Forschung von realen Implementierungszeitschienen bemängelt [39]. In Abhängigkeit davon, ob Implementierungswissenschaft begleitend, unterstützend oder erklärend in Forschungsvorhaben eingebunden ist [15], kann es zu unterschiedlichen Anforderungen an die umsetzenden Organisationen und somit zu einer Mehrbelastung in ressourcenschwachen Kontexten kommen. Im angloamerikanischen Raum wird Kritik an fehlender oder mangelnder Equity-Orientierung, also der Beachtung von Gerechtigkeitsthematiken in der Implementierungswissenschaft, geäußert [39, 40]. Im deutschsprachigen Raum werden hingegen entsprechende Themen überhaupt nicht diskutiert, was als Hinweis gedeutet werden kann, dass eine kritische Auseinandersetzung mit der Implementierungswissenschaft und ihrem Potenzial für Public Health bislang größtenteils fehlt. Darüber hinaus wird international die empirielastige und positivistische, normative Ausrichtung kritisiert, die erkenntnistheoretisch in der evidenzbasierten Medizin und der Logik der traditionellen Schulmedizin fußt, welche komplexe und voneinander abhängende Prozesse und Systeme in Einzelteile bricht [41]. Dies birgt die Gefahr, dass systemimmanente Widersprüche nicht ausreichend einbezogen werden. Zudem werden konstruktivistische und interpretative Ansätze sowie kritische Theorie nicht hinreichend berücksichtigt [41]. Dies grenzt die Implementierungswissenschaft vom Themengebiet der Gesundheitssystemforschung (Health Policy and Systems Research) ab, in dem z. B. die Analyse von Machtverhältnissen in soziotechnischen, komplex adaptiven Systemen oft stärker im Fokus steht [42].

## Potenziale der Implementierungswissenschaft

Die Implementierungswissenschaft unterstützt die Integration von wissenschaftlichen Erkenntnissen in die Praxis durch die systematische Erhebung von Faktoren, die die Umsetzung ermöglichen oder behindern [8, 9]. Für die strukturierte Erforschung dieser Einflussfaktoren steht eine Reihe flexibler TMFs zur Verfügung, die die systematische Erfassung aller Aspekte der Implementierung auch bei komplexen und interagierenden Interventionen im Bereich der Individual- und Bevölkerungsgesundheit unterstützen können. Daher liegt es vor dem Hintergrund der oftmals diskutierten Umsetzungslücke für evidenzbasierte präventive und Versorgungsinterventionen nahe, trotz der o. g. Kritikpunkte die Implementierungswissenschaft in Deutschland methodisch und inhaltlich weiter zu stärken [43].

Als Forschungsgebiet, das die Art der Implementierung von Public-Health-Interventionen, einschließlich verhältnispräventiver Ansätze, betrachtet, bietet die Implementierungswissenschaft zudem wertvolle Möglichkeiten, Erkenntnisse zu gewinnen zur erfolgreichen Umsetzung von Maßnahmen, die die Verringerung sozioökonomischer Ungleichheit und die Verbesserung gesundheitlicher Chancengerechtigkeit zum Ziel haben. Hierzu muss die Implementierungswissenschaft jedoch einen expliziten Equity-Fokus einnehmen und die Implementierungsprozesse und -outcomes mit einer solchen Linse untersuchen. Dies ist insbesondere deshalb von Bedeutung, weil Public-Health-Interventionen selbst Ungleichheiten generieren können [44], z. B. durch die unreflektierte Implementierung populationsbasierter Maßnahmen im Rahmen von Informationskampagnen [44] oder Digitalisierungsmaßnahmen [45]. Im angloamerikanischen Raum wurde dieses Potenzial der Implementierungswissenschaft, Versorgungsungleichheiten zu verringern, in den vergangenen Jahren zunehmend erkannt und erfolgreich genutzt [46–48].

Implementierungsforschung kann in formativer Hinsicht Evidenz bieten, die zur Verbesserung des Interventionsdesigns, zur Vermeidung von Barrieren oder des häufig zu beobachtenden Intensitätsabfalls der Effektivität von Maßnahmen in Alltagskontexten über die Zeit („Voltage Drop“ [46]) beiträgt. Auch das Thema Nichtinanspruchnahme sowie Fragen zur Verstetigung und Nachhaltigkeit von Interventionen oder zur Deimplementierung können bearbeitet werden.

Schließlich kann durch eine explizite Forschungsausrichtung die Identifikation unerwünschter Effekte von Interventionen [49] in den Blick genommen werden, womit die Brücke zu systemischem Denken geschlagen und so die Komplexität sowohl der Interventionen als auch der sie umgebenden Systeme berücksichtigt werden kann.

## Fazit

Ungenutzte Potenziale für Implementierungswissenschaft zu mobilisieren, kann dazu beitragen, evidenzbasierte Interventionen zur gesundheitlichen Versorgung sowie zur Gesundheitsförderung und Prävention gezielter und nachhaltiger umzusetzen, aus den Prozessen zu lernen, Chancengerechtigkeit zu erhöhen und damit Public Health in Deutschland zu stärken.

## Supplementary Information

Englische Version des Artikels

## Footnotes

1 https://www.bewegung-bei-krebs.org/bbk (Zugriff am 28.01.2025).

2 https://move-onko.de/ (Zugriff am 28.01.2025).

3 https://itcproject.org/ (Zugriff am 28.01.2025).

4 https://www.fruehehilfen.de/forschung-im-nzfh/implementierungsforschung/ (Zugriff am 28.01.2025).

5 https://www.klinikum.uni-heidelberg.de/personen/prof-dr-michel-wensing-7511 (Zugriff am 28.01.2025).

6 https://www.uni-bremen.de/institut-fuer-public-health-und-pflegeforschung/abteilungen-arbeitsgruppen/pflegeforschung/pflegewissenschaftliche-evaluations-und-implementierungsforschung, https://www.bips-institut.de/en/the-institute/departments/prevention-and-evaluation/implementation-research-and-mental-health.html (Zugriff am 28.01.2025).

7 https://www.foga-foerderprogramm.de/DE/GefoerderteVorhaben/Projektsteckbrief-Professur-13FG0003.html (Zugriff am 28.01.2025).

8 https://implementation.eu/training-and-education/ (Zugriff am 28.01.2025).

9 https://www.uni-heidelberg.de/de/studium/alle-studienfaecher/versorgungsforschung-und-implementierungswissenschaft-im-gesundheitswesen/versorgungsforschung-und-implementierungswissenschaft-im-gesundheitswesen-master (Zugriff am 28.01.2025).

10 https://www.fliedner-fachhochschule.de/zertifikatsstudium/evidenzbasierte-gesundheitsversorgung-mas/implementierungsmanager-healthcare/ (Zugriff am 02.03.2025).

11 https://www.hs-osnabrueck.de/module/22m0901/. https://www.uni-luebeck.de/index.php?id=12828&tx_webparser_pi1%5Bmodulid%5D=2405 (Zugriff am 28.01.2025).

12 https://dnvf.de/ver%C3%B6ffentlichungen/dnvf-journal.html (Zugriff am 28.01.2025).
