# Supplementary material for: Status, classification, and potential of implementation research: an inventory for public health in Germany
Source: Bundesgesundheitsblatt Gesundheitsforschung Gesundheitsschutz. 2025 Jun 30;68(7):718–27. [Article in German] doi: 10.1007/s00103-025-04077-7 (PMC12254172; doi:10.1007/s00103-025-04077-7)
Supplement: Supplementary file 1 — Englische Version des Artikels [file 103_2025_4077_MOESM1_ESM.docx]

This is an English translation of the article: "Stand, Einordnung und Potential der Implementierungswissenschaft: eine Bestandsaufnahme für Public Health in Deutschland" (Bundesgesundheitsblatt 7/2025). The responsibility for the translation lies solely with the authors. Please note that only the original German-language article can be cited.

**Status, positioning and potential of implementation science: a situation analysis for public health in Germany**

Heide Weishaar^1^, Anna Kuehne^2^, Kayvan Bozorgmehr^3^, Hajo Zeeb^4,5^

The authors H. Weishaar and A. Kuehne share first authorship.

^1^ Robert Koch Institute, Berlin, Germany

^2^ Chair for Public Health, Centre for Evidence-Based Health Care, University Hospital and Medical Faculty Carl Gustav Carus of the Technical University Dresden, Dresden, Germany

^3^ Department of Population Medicine and Health Services Research, School of Public Health, Bielefeld University, Bielefeld, Germany

^4^ Leibniz Institute for Prevention Research and Epidemiology BIPS, Bremen, Germany

^5^ Health Sciences Bremen, University of Bremen, Germany

**Correspondence:**

Dr. Heide Weishaar
Robert Koch-Institut
Gerichtstr. 27
13347 Berlin
Deutschland
[weishaarh@rki.de](mailto:weishaarh@rki.de)

**Status and potential of implementation research: an inventory for public health in Germany**

# This is an English translation of the article: 'Stand, Einordnung und Potential der Implementierungswissenschaft: eine Bestandsaufnahme für Public Health in Deutschland' (Bundesgesundheitsblatt 7/2025). The responsibility for the translation lies solely with the authors. Please note that only the original German-language article can be cited.

***Abstract***

The implementation of high-quality, evidence-based practice to improve the health of populations is a prerequisite and a core element of public health. However, gaps often exist between research and practice, leading to the underuse of effective interventions and inappropriate use on non-effective interventions. Implementation science aims to bridge the gap between knowledge production and implementation by generating evidence to improve the transfer of research findings and evidence-based practices into routine action. This discussion article reflects on the state of implementation science in Germany. First, we provide a descriptive classification of implementation science, followed by a review of selected theories, models and frameworks that are used in implementation research to systematically analyze implementation strategies, barriers, facilitators and outcomes of interventions. Subsequently, implementation science in Germany is examined with regard to specific projects, institutional integration, teaching, and networking among experts. The article concludes with a critical reflection on the limitations and potential of implementation science for public health in Germany.

An English full-text version of this article is available at SpringerLink as Supplementary Information.

**Keywords***:* implementation science; implementation science theories, models and frameworks (TMFs); Germany; public health

**What is implementation science?**

For some years now, implementation science has been establishing itself internationally and nationally as an independent field of research that is closely related to health services research in particular. The aim of this article is to highlight and critically classify topics and concepts of implementation science, to outline - without claiming to be exhaustive - the status of implementation science in Germany and to discuss future directions and development potential.

Medical, epidemiological and public health research provides insights into how certain individual, organizational or structural influencing factors should be changed or how measures should be designed in order to improve healthcare or public health. The focus is often on the effectiveness under specific conditions and on certain pre-selected groups of people. Much less is known about the implementation of measures and programs under everyday conditions, which usually differs significantly from implementation in more controlled research settings. This often leads to low external validity and implementation.

The limited integration of evidence results, among other things, in long implementation times: The implementation of interventions in the medical context takes on average 17 years [1]. In the public health context, the implementation of population-based interventions can take even longer, as shown by the several decades of development and sometimes patchy implementation of effective tobacco control programs [2, 3]. Furthermore, due to a lack of evaluation and scientific monitoring of measures and interventions, the de-implementation of measures that do not have the desired effects is rare or delayed [4]. The lack and delay in implementing effective and de-implementing ineffective interventions contribute to the underuse of innovative, demonstrably effective interventions and to the mis- and overuse of ineffective interventions; high-quality evidence-based interventions are not used or not used effectively [5]. This is where implementation research comes in: It is aimed at reducing the gap between knowledge production and implementation by means of multi- and interdisciplinary research using, for example, qualitative and quantitative methods from the social and health sciences, epidemiology and other scientific disciplines.

Implementation science encompasses implementation research in the field of health care, population health and health policy [6]. The definition of implementation research includes the following aspects:

(a) implementation research is concerned with implementation strategies to promote the systematic integration of research findings and evidence-based practices into routine action; thus, it contributes to improving the quality and effectiveness of health services [1, 6, 7];

b) Implementation research focuses on the complex issues and challenges related to the practical implementation of innovations and regularly includes questions of sustainability and capacity building [8, 9];

c) Implementation research is interested in implementation-related outcome parameters such as acceptability, adoption, feasibility, appropriateness, cost, fidelity, penetration and sustainability [9].

Proving the effectiveness of interventions is not the central topic of implementation research, but a prerequisite of this type of research. The aim is to obtain concrete, comprehensive findings on the implementation of health-related interventions under often widely varying conditions in different contexts and for diverse target groups. This also includes research on de-implementation, i.e. the termination of measures that are part of established routines of action but are ineffective in real life or even associated with negative consequences [10].

**How does implementation science complement other fields of research?**

Implementation research is used in a variety of academic disciplines to systematically analyse the complex interactions between interventions and their environment. Corresponding analyses are also carried out in health services research and public health research [8, 11, 12, 13] (Fig. 1). A statement issued by the German Research Foundation (DFG) in 2008 describes health services research as a discipline that focuses more on patients or those at risk of illness and on medical measures, while public health is primarily concerned with maintaining health, with focus on non-medical measures [14]. Following the logic outlined above, implementation research in public health encompasses research into implementation strategies, influencing factors and implementation outcomes in relation to complex public health measures with the aim of promoting health [15]. Implementation research in health services research, on the other hand, concentrates on medical interventions with a focus on the health of the individual and on early detection, curative medical care and rehabilitation [16, 17]. However, implementation research – in contrast to health service research – does not include the research fields of medical needs assessment, health systems analyses or basic effectiveness evaluation, which form part of the research portfolio of health services research [16, 17, 18], nor does it include further research areas in public health. In addition, implementation research design places great emphasis on the participation of stakeholders who are relevant to the respective intervention [19, 20, 21]. Furthermore, several models and frameworks of implementation research focus more strongly on the aspect of acceptability (among implementers and users) and the fit of the intervention as well as the sustainability of the implementation [17, 18].

**Implementation science theories, core concepts, models and frameworks**

Implementation science primarily focuses on gaining insights that make the integration of research results into practice more likely or more effective, or that enable interventions to be improved and adapted, thus reducing the know-do-gap. It deals comprehensively with the question of which influencing factors promote or inhibit the implementation of measures and which implementation strategies can be conducive to implementation. Accordingly, result parameters are usually examined in implementation science and are referred to as implementation outcomes.

As indicators for the success of implementation, implementation outcomes act as mediators between an intervention and its success, because an intervention can only be successful if it is adequately implemented [9]. In implementation science, a distinction is made between the effectiveness of the *implementation* and the effectiveness of the *intervention*. If the implementation of research results does not work because the intervention is inappropriate, this is referred to as an intervention error; if the implementation fails because an appropriate intervention was implemented incorrectly, this is referred to as an implementation error.

Implementation research also focuses on adherence to the specifications of an intervention with regard to the accuracy of implementation or fidelity [9]. Here, attention is paid to implementation quantity (i.e. the completeness of the intervention) and implementation quality (i.e. the accuracy of the implementation of the intervention). However, changes in the implementation of interventions may be necessary in order to adapt the intervention to the specific setting.

In order to identify influencing factors and evaluate result parameters, it is helpful to systematically monitor and analyze them. In implementation science, theories, models and frameworks (TMFs) are used to support these systematic analyses. TMFs help to select or develop implementation strategies, to describe factors influencing implementation and to measure outcomes. A large number of TMFs now exist, some of which have overlaps and synergies [22]. A review of dissemination and implementation research by Tabak et al. from 2012 identifies a total of 61 TMFs [22]. The overview is practically oriented and aims to make it easier for researchers to select suitable constructs for their own research based on central categories (focus on dissemination or implementation, construct flexibility, socio-ecological level). A later review by Nilsen [23] proposes a taxonomy of TMFs that goes beyond the organizing principles of Tabak et al [22]. According to Nilsen's taxonomy, TMFs in implementation science serve three main purposes:

1) describing and/or guiding the integration from research to practice,

2) understanding/explaining factors influencing implementation outcomes, and

3) the evaluation of implementation.

Nilsen assigns five different TMF categories to these three goals, such as process models to the first goal, determinant models and classical theories to the second goal. By way of example, two established frameworks are briefly explained here, which relate to the contexts in which health-related innovations are implemented.

The “Consolidated Framework for Implementation Research” (CFIR; [11, 13]) offers a comprehensive approach that draws on classical theories from sociology and psychology in order to systematically record environmental aspects of interventions – so-called contextual determinants. The identified facilitators and barriers can then be considered in the alignment and adaptation of implementation strategies. The CFIR, which has now been translated into German, has five basic constructs [24], each of which is explained with detailed criteria and precise descriptions, thus facilitating the application of the framework. The constructs are: Innovation, Outer setting, Inner setting, Individuals, and Implementation process. Some of these domains were adapted and expanded in a revision of the framework [13], e.g. outcomes were included. The authors emphasize that not all domains are of equal importance for every intervention and implementation and that the CFIR should be used flexibly.

The “Context and Implementation of Complex Interventions” (CICI) framework focuses on the implementation and context of complex interventions [12]. It justifies this by stating that the surrounding conditions are of the utmost importance for complex interventions. The CICI framework systematically draws on predecessor models, including the CFIR, and analyzes them with regard to the special features of complex interventions. In the CICI framework, different domains are specified for the three dimensions of context, implementation and setting, which enable a differentiated allocation of specific intervention components and thus make the interaction between different components and the respective setting transparent. The CICI framework uses a checklist to encourage systematic reflection on the geographical, epidemiological, socio-cultural, economic, ethical, legal and political contexts in relation to the respective intervention. Pfadenhauer et al. demonstrate the application and the knowledge gained from the framework using a concrete example, the implementation of measures to improve air quality in Ireland [12].

To measure the success of implementation processes, Proctor's taxonomy [25] focuses specifically on the evaluation of implementation outcomes. It emphasizes different dimensions that are important to evaluate the adoption and use of interventions in practice. The domains considered are acceptability, adoption, feasibility, appropriateness, cost, fidelity, penetration and sustainability [25].

When considering the use of adequate frameworks, it is important to critically examine their alignment with one's own research object [22]. CFIR aims to identify influencing factors and has a strong contextual focus: it provides a pragmatic structure for a more precise understanding of implementation processes and outcomes and focuses on individual actors (e.g. their motivation and competencies) [11]. When it comes to complex public health interventions and the interaction of different aspects with settings, CICI may be appropriate, especially when working across settings [12]. With regard to a precise operationalization of outcome parameters, Proctor's taxonomy of implementation outcomes can be consulted: It uses clear metrics that can be operationalized qualitatively or quantitatively and applied to different sectors (health care, education, social services) [25].

**How is implementation science represented in Germany?**

Implementation science can be found in many areas of public health and health services research. In the following, we outline the state of implementation science in Germany, without claiming to be exhaustive and while recognizing that the above-mentioned elements and aspects of implementation science may be represented in German research beyond the areas mentioned below. First, we present four examples from the German-speaking context to illustrate the diversity and breadth of implementation research in the health sector.

**Sport and exercise therapy for cancer patients.** In two large collaborative research projects funded by German Cancer Aid, interdisciplinary research teams from different locations investigate how quality-assured sport and exercise therapy can be implemented more widely for cancer patients in Germany. The effectiveness of sport and exercise therapy has been proven for many years in terms of improving quality of life and a number of other outcomes [26] but has so far been insufficiently used in routine oncological care. The projects IMPLEMENT^[[1]](#footnote-1)^ and MOVE-ONKO^[[2]](#footnote-2)^ are now using implementation science methods to investigate which barriers and facilitators influence different implementation strategies and how these can be considered in specific pilot projects in order to later promote the nationwide implementation of quality-assured sport and exercise therapy for cancer patients.

**Tobacco control.** Research on tobacco control measures and in particular the International Tobacco Control (ITC) Policy Evaluation Project^[[3]](#footnote-3)^ are examples of implementation research. The ITC project is led by scientists from the University of Waterloo in Canada; the German Cancer Research Center is involved as a German cooperation partner. The aim of the international cohort study on tobacco consumption is the systematic and comprehensive evaluation of various tobacco prevention and tobacco control measures^8^. This includes the evaluation of policy interventions (e.g. smoke-free legislation, price and tax policies, legally regulated warning labels) and the identification of factors that influence effective tobacco control policies^8^.

**Operational research of the National Center for Early Interventions**. The applied research of the National Center for Early Interventions’ provides an example of implementation research in public health in Germany^[[4]](#footnote-4)^. As part of a process evaluation, the National Center for Early Interventions regularly collects data on the structural development and establishment of support during the early years of life and analyzes its implementation, e.g. with regard to formal responsibilities, the embeddedness of early years support in municipal planning and management, as well as equipment and quality features of early years support^12^. The implementation of early years support in everyday practice is evaluated using specific indicators^12^.

**Implementation research with a focus on public health services.** Implementation research can make a significant contribution to the successful implementation of new evidence-based measures in the Public Health Service. While the number of scientifically published project-related evaluations and applied research projects in the field of health promotion, prevention and care in the public health service has increased significantly over the past 20 years, systematic approaches to implementation research are still rare [27, 28, 29, 30]. Intra- and after-action reviews (a method of adapting and sustainably improving crisis management in the public health service after a crisis and in preparation for future crises [31, 32]), are examples of the first systematic approaches that show similarities to implementation research with regard to the structured recording of success factors [28, 33, 34].

A study by Schultes et al. [35] identifies the following seven barriers and facilitating factors for conducting implementation science in German-speaking countries:

(i) the characteristics of implementation science as a scientific discipline, including a lack of a common understanding of what can be called implementation science;

(ii) the conditions and particular challenges for corresponding research projects;

(iii) personal factors related to implementation;

(iv) the (limited) networking between implementation scientists;

(v) the (limited) opportunities for skills acquisition;

(vi) the (insufficient) financial support for implementation research; and

(vii) the (limited) willingness to establish implementation science in a scientific context.

In line with the last point, implementation science in Germany is not yet broadly anchored institutionally compared to the Anglo-American region [35]. Only a few scientific institutions pay explicit attention to the topic. There is only one designated professorship for health services research and implementation science at the University of Heidelberg^[[5]](#footnote-5)^. There are two professorships at the University of Bremen that deal with implementation science in two specialized areas of health^[[6]](#footnote-6)^, and at the beginning of 2025, an endowed professorship related to implementation science was established at Leipzig University^[[7]](#footnote-7)^. In addition, there are individual academic institutions that focus on implementation science as part of research or working groups. Implementation science is particularly common at universities of applied sciences, which is presumably related to their focus on application.

A Europe-wide database of the European Implementation Collaborative provides an overview of further training and courses in implementation science in Europe^[[8]](#footnote-8)^. Most trainings and courses are offered as part of degree programs, e.g. master's and doctoral programs; however, there are also a number of courses for professional development^9^. In Heidelberg, a master's degree program is dedicated to health services research and implementation science in the healthcare sector^[[9]](#footnote-9)^. The Fliedner University of Applied Sciences offers a part-time certificate course for healthcare implementation managers^[[10]](#footnote-10)^. In addition, a pragmatic online search shows that some teaching in implementation science in Germany is located at universities of applied sciences or within degree programs for healthcare professions^[[11]](#footnote-11)^.

As the above overview shows, implementation science has yet to become widely established in the academic teaching and research landscape in Germany [35]. Obstacles include the fact that the terminology, which has mainly established itself in English-speaking countries, is only partially transferable to German-speaking countries [35]. Financial support for implementation research^[[12]](#footnote-12)^ usually takes place within the framework of overarching funding programs such as the innovation fund of the Federal Joint Committee of the health insurance funds (G-BA), and this focuses mainly on health services research projects in the narrower sense. Due to their focus on basic research, other funding formats, such as those of the German Research Foundation, have so far offered little room for the issues and methods as well as the real-world relevance of implementation research. Although existing funding programs increasingly emphasize the need to integrate research into practice, there is only limited explicit funding for research projects that investigate influencing factors and implementation strategies.

The networking and exchange of scientists is also of great importance for the research field of implementation science. National and international networks and scientific journals focus on implementation science. In addition to several international journals that focus on implementation science, the German Network for Health Services Research (DNVF) published the journal *Health Care Research & Implementation* as a supplement to the journal “Das Gesundheitswesen”^[[13]](#footnote-13)^. Of course, implementation science topics are not only found in specific journals, but also in a variety of journals from medical, public health and other fields. An international analysis of publications in implementation science shows that the identification of relevant literature is complicated by the unspecific and varying terminology that is spread across several disciplines [36]. Table 1 lists relevant networks and publication organs in the field of implementation science without claiming to be exhaustive.

**Criticism of implementation science**

Despite strong voices emphasizing the benefits of implementation science, the research field also faces criticism. One point of criticism is that the sheer number of TMFs, terminology and approaches has led to fragmentation and siloing of the field [37], which prevents integrative findings. In addition, the lack of practical relevance of research [38, 39] and the decoupling of research from real implementation timelines are criticized [39]. Depending on whether implementation science is involved in research projects in an operational, supporting or explanatory capacity [15], this can lead to different demands on the implementing organizations and thus to an additional burden in resource-poor contexts. In the Anglo-American context, the lack of or insufficient equity orientation in implementation science is criticised [39, 40]. In German-speaking countries, on the other hand, such topics are not discussed at all, which can be interpreted as an indication that a critical reflection on implementation science and its potential for public health has so far been largely lacking. In addition, there is international criticism of the empiricist and positivist, normative orientation, which is epistemologically rooted in evidence-based medicine and the logic of traditional orthodox medicine, which breaks complex and interdependent processes and systems into individual parts [41]. This bears the risk that contradictions that are inherent in the system are not sufficiently considered. Constructivist and interpretative approaches as well as critical theory are also not sufficiently taken into account [41]. This distinguishes implementation science from the field of health policy and systems research, which focuses more, for example, on the analysis of power relations in socio-technical, complex-adaptive systems [42].

**The potential of implementation science**

Implementation science supports the integration of scientific knowledge into practice by systematically identifying factors that enable or hinder implementation [8, 9]. A number of flexible TMFs are available for the structured investigation of these influencing factors, which can support the systematic recording of all aspects of implementation, even for complex and interacting interventions in the field of individual and population health. Against the backdrop of the frequently discussed implementation gap for evidence-based preventive and health care interventions, it therefore makes sense to further strengthen implementation science in Germany in terms of methodology and content, despite the above-mentioned points of criticism [43].

As a field of research that looks at the nature of implementation of public health interventions, including behavioral prevention approaches, implementation science also offers valuable opportunities to gain insights into the successful implementation of interventions that aim to reduce socioeconomic inequalities and improve health equity. To do so, however, implementation science must adopt an explicit equity focus and examine implementation processes and outcomes through such a lens. This is particularly important because public health interventions themselves can generate inequalities [44], e.g. through the unreflected implementation of population-based measures as part of information campaigns [44] or digitization measures [45]. In the Anglo-American region, this potential of implementation science to reduce health inequalities has been increasingly recognized and successfully used in recent years [46, 47, 48].

Implementation research can provide formative evidence that contributes to improving the intervention design, avoiding barriers or the frequently observed drop in the effectiveness of interventions in everyday contexts over time (“voltage drop” [46]). The topic of non-utilization as well as questions regarding the continuation and sustainability of interventions or de-implementation can also be addressed.

Finally, the identification of unintended effects of interventions [49] can be addressed through an explicit research orientation, thus building a bridge to systems thinking and taking into account both the complexity of the interventions and the surrounding systems.

**Conclusion**

Mobilizing unused potential for implementation science can help to implement evidence-based interventions for health care, health promotion and prevention in a more targeted and sustainable way, learn from the underlying processes, increase equal opportunities and thus strengthen public health in Germany.

**Acknowledgments:** We thank Marie-Therese Schultes for her collegial comments on the article.

**Compliance with ethical guidelines**

Conflict of interest: Heide Weishaar, Anna Kühne, Kayvan Bozorgmehr and Hajo Zeeb declare that they have no conflict of interest.

No studies on humans or animals were conducted by the authors for this article. The ethical guidelines apply to the studies listed.

# *References*

1. Rubin R (2023) It Takes an Average of 17 Years for Evidence to Change Practice—the Burgeoning Field of Implementation Science Seeks to Speed Things Up. JAMA 329:1333-1336. <http://doi.org/10.1001/jama.2023.4387>

2. Yach D (2014) The origins, development, effects, and future of the WHO Framework Convention on Tobacco Control: a personal perspective. The Lancet 383:1771-1779. <https://doi.org/10.1016/S0140-6736(13)62155-8>

3. Gravely S, Giovino GA, Craig L et al. (2017) Implementation of key demand-reduction measures of the WHO Framework Convention on Tobacco Control and change in smoking prevalence in 126 countries: an association study. The Lancet Public Health 2:e166-e174. <http://doi.org/10.1016/S2468-2667(17)30045-2>

4. Virginia M, Callie W-B, Sara M, Collin M, Daniel J N (2023) Missing the Target: Mis-implementation and De-implementation. In: Ross C B, Graham A C, Enola K P (eds) Dissemination and Implementation Research in Health. p 251-268

5. Institute of Medicine (2001) Crossing the Quality Chasm: A New Health System for the 21st Century. The National Academies Press, Washington, DC

6. Eccles MP, Mittman BS (2006) Welcome to Implementation Science. Implementation Science 1:1. <https://doi.org/10.1186/1748-5908-1-1>

7. Rapport F, Clay Williams R; Churruca K; Hogden A; Braithwaite J (2017) The struggle of translating science into action: Foundational concepts of implementation science. Evaluation in Clinical Practice. <https://doi.org/10.1111/jep.12741:117-126>. <https://doi.org/10.1111/jep.12741>

8. Stummer FO (2023) Einleitung – Was ist Implementation Science? In: Implementierungsstrategien im Gesundheitswesen: Die 50 wichtigsten Implementierungs-Frameworks in der Praxis - eine Literaturrecherche. Springer Fachmedien Wiesbaden, Wiesbaden, p 1-11

9. Franz P (2014) Implementationsforschung: Grundbegriffe und Konzepte. Hogrefe 65:122–128. <https://doi.org/10.1026/0033-3042/a000214>

10. Vollmar HC, Santos S, de Jong A, Meyer G, Wilm S (2017) Wie gelangt Wissen in die Versorgung? Bundesgesundheitsbl. 60:1139-1146. <https://doi.org/10.1007/s00103-017-2612-z>

11. Damschroder LJ, Aron DC, Keith RE, Kirsh SR, Alexander JA, Lowery JC (2009) Fostering implementation of health services research findings into practice: a consolidated framework for advancing implementation science. Implementation Science 4:50. <https://doi.org/10.1186/1748-5908-4-50>

12. Pfadenhauer LM, Gerhardus A, Mozygemba K et al. (2017) Making sense of complexity in context and implementation: the Context and Implementation of Complex Interventions (CICI) framework. Implement. sci. 12:21. <https://doi.org/10.1186/s13012-017-0552-5>

13. Damschroder LJ, Reardon CM, Widerquist MAO, Lowery J (2022) The updated Consolidated Framework for Implementation Research based on user feedback. Implement. sci. 17:75. <https://doi.org/10.1186/s13012-022-01245-0>

14. Hoffmann F, Gerhardus A, Härter M (2024) Versorgungsforschung und die Bezüge zu Public Health. In: Pfaff H, Neugebauer EAM, Ernstmann N, Härter M, Hoffmann F (eds) Versorgungsforschung: Theorien – Methoden – Praxis. Springer Fachmedien Wiesbaden, Wiesbaden, p 31-38

15. Wensing M, Wilson P (2023) Making implementation science more efficient: capitalizing on opportunities beyond the field. Implement. Scien. 18:40. <https://doi.org/10.1186/s13012-023-01298-9>

16. Wensing M, Ullrich C (2023) Foundations of Health Services Research. Springer Nature, Cham

17. Ständige Kongresskommission Deutscher Kongress für Versorgungsforschung (2003) Memorandum zur Versorgungsforschung in Deutschland. Situation – Handlungsbedarf – Strategien. In:<https://dnvf.de/files/theme_files/pdf/PDF-Publikationen/1.%20Memorandum%202003.pdf>. Zugegriffen: 10. April 2025. Zugegriffen:

18. Bundesärztekammer. Arbeitskreis Versorgungsforschung beim Wissenschaftlichen Beirat (2004) Definition und Abgrenzung der Versorgungsforschung. In:<https://www.bundesaerztekammer.de/fileadmin/user_upload/_old-files/downloads/pdf-Ordner/Versorgungsforschung/Definition.pdf> Zugegriffen:

19. Pérez Jolles M, Willging CE, Stadnick NA et al. (2022) Understanding implementation research collaborations from a co-creation lens: Recommendations for a path forward. Front Health Serv. 2<https://doi.org/10.3389/frhs.2022.942658>

20. Estabrooks PA, Brownson RC, Pronk NP (2018) Dissemination and Implementation Science for Public Health Professionals: An Overview and Call to Action. Prev Chronic Dis 15:E162. <https://doi.org/10.5888/pcd15.180525>

21. Lobb R, Colditz GA (2013) Implementation science and its application to population health. Annu Rev Public Health 34:235-251. <https://doi.org/10.1146/annurev-publhealth-031912-114444>

22. Tabak RG, Khoong EC, Chambers DA, Brownson RC (2012) Bridging research and practice: models for dissemination and implementation research. Am J Prev Med 43:337-350. <https://doi.org/10.1016/j.amepre.2012.05.024>

23. Nilsen P (2015) Making sense of implementation theories, models and frameworks. Implement. Scien. 10:53. <https://doi.org/10.1186/s13012-015-0242-0>

24. Regauer V, Seckler E, Campbell C et al. (2021) German translation and pre-testing of Consolidated Framework for Implementation Research (CFIR) and Expert Recommendations for Implementing Change (ERIC). Implement. Scien. 2:120. <https://doi.org/10.1186/s43058-021-00222-w>

25. Proctor E, Silmere H, Raghavan R et al. (2011) Outcomes for implementation research: conceptual distinctions, measurement challenges, and research agenda. Adm Policy Ment Health 38:65-76. <https://doi.org/10.1007/s10488-010-0319-7>

26. Baumann FT, Jensen W, Berling-Ernst A, Theurich S, Leitzmann M, Götte M (2024) Exercise Therapy in Oncology—the Impact on Quality of Life and Side Effects. Dtsch Arztebl Int 121:331-337. <https://doi.org/10.3238/arztebl.m2024.0038>

27. Scheffler A, Klocker L, Puls A, Hummers E, Demmer I (2024) [Facilitators and barriers to the implementation of health promotion in daycare centers and elementary schools based on four selected projects]. Bundesgesundheitsbl. 67:1021-1030. <https://doi.org/10.1007/s00103-024-03935-0>

28. Höglund-Braun H, Quartey AL, Ganter M et al. (2024) An After-Action Review at Municipal Level in the Public Health Service - Lessons Learned at the Corona Unit of the Duesseldorf Public Health Authority after the end of the Covid-19 Pandemic, March 2023. Gesundhwes. 86:769-775. <https://doi.org/10.1055/a-2329-7058>

29. Hentrich SAM, Lenkowski M, Seebaß K, Ottmann S, John D (2024) Dezentrale Gesundheitsförderung nach dem Präventionsgesetz in Nürnberg: Evaluationsergebnisse und Erfahrungen aus dem Projekt „Gesundheit für alle im Stadtteil“. Gesundhwes. 86:103-110. <https://doi.org/10.1055/a-2206-1612>

30. Schütze D, Engler F, Nohl-Deryk P, Müller B, Müller A (2022) Implementing a secure instant messaging app in the COVID-19 pandemic: Usage experiences of primary care physicians and local health authorities. Z. Evid. Fortbild. Qual. Gesundhwes. 173:40-48. <https://doi.org/10.1016/j.zefq.2022.04.011>

31. Robert Koch Institute (2025) FG 31: ÖGD-Kontaktstelle. Krisenmanagement, Ausbruchsuntersuchungen und Trainingsprogramme. <https://www.rki.de/DE/Institut/Organisation/Abteilungen/Abteilung-3/FG31/fg31_org.html?templateQueryString=after+action+reviews+preparedness>. Zugegriffen: 23 April 2025

32. Akademie für Öffentliches Gesundheitswesen (2025) ILEAS. <https://www.akademie-oegw.de/die-akademie/projekte/ileas>. Zugegriffen: 23 April 2025

33. Stoto MA, Nelson C, Piltch-Loeb R, Mayigane LN, Copper F, Chungong S (2019) Getting the most from after action reviews to improve global health security. Glob. Health 15:58. <https://doi.org/10.1186/s12992-019-0500-z>

34. Sarma N, Seidel J, Hommes F et al. (2024) Prekäre Wohn- und Arbeitsverhältnissen in der COVID-19-Pandemie. Ergebnisse eines After-Action-Review in Deutschland, 2020-2022. Gesundhwes. 86:S81. <https://doi.org/10.1055/s-0044-1781813>

35. Schultes M-T, Finsterwald M, Brunkert T, Kien C, Pfadenhauer L, Albers B (2022) Barriers and Facilitators for Conducting Implementation Science in German-Speaking Countries: Findings from the Promote ImpSci Interview Study. Glob. Implement. Res. Appl. 2:120-131. <https://doi.org/10.1007/s43477-022-00046-3>

36. Mielke J, Brunkert T, Zullig LL et al. (2021) Relevant Journals for Identifying Implementation Science Articles: Results of an International Implementation Science Expert Survey. Front. Public Health 9:639192. <https://doi.org/10.3389/fpubh.2021.639192>

37. Chambers DA, Emmons KM (2024) Navigating the field of implementation science towards maturity: challenges and opportunities. Implement. Scien. 19:26. <https://doi.org/10.1186/s13012-024-01352-0>

38. Harvey G, Rycroft-Malone J, Seers K et al. (2023) Connecting the science and practice of implementation - applying the lens of context to inform study design in implementation research. Front. Health Serv. 3:1162762. <https://doi.org/10.3389/frhs.2023.1162762>

39. Beidas RS, Dorsey S, Lewis CC et al. (2022) Promises and pitfalls in implementation science from the perspective of US-based researchers: learning from a pre-mortem. Implement. Scien. 17:55. <https://doi.org/10.1186/s13012-022-01226-3>

40. Shelton RC, Brownson RC (2024) Enhancing Impact: A Call to Action for Equitable Implementation Science. Prev. Sci. 25:174-189. <https://doi.org/10.1007/s11121-023-01589-z>

41. Boulton R, Sandall J, Sevdalis N (2020) The Cultural Politics of ‘Implementation Science’. J. Med. Humanit. 41:379-394. <https://doi.org/10.1007/s10912-020-09607-9>

42. Sheikh K, George A, Gilson L (2014) People-centred science: strengthening the practice of health policy and systems research. Health Res. Policy Syst. 12:19. <https://doi.org/10.1186/1478-4505-12-19>

43. Hollederer A, Wildner M (2015) Versorgungsforschung für den Öffentlichen Gesundheitsdienst (ÖGD) und das öffentliche Gesundheitswesen. Gesundhwes. 77:232-236. <https://doi.org/10.1055/s-0034-1390410>

44. Lorenc T, Petticrew M, Welch V, Tugwell P (2013) What types of interventions generate inequalities? Evidence from systematic reviews. J. Epidemiol. Community Health 67:190-193. <https://doi.org/10.1136/jech-2012-201257>

45. Veinot TC, Mitchell H, Ancker JS (2018) Good intentions are not enough: how informatics interventions can worsen inequality. J. Am. Med. Inform. Assoc. 25:1080-1088. <https://doi.org/10.1093/jamia/ocy052>

46. Ramanadhan S, Alemán R, Bradley CD et al. (2024) Using Participatory Implementation Science to Advance Health Equity. Annu. Rev. Public Health 45:47-67. <https://doi.org/10.1146/annurev-publhealth-060722-024251>

47. Gustafson P, Abdul Aziz Y, Lambert M et al. (2023) A scoping review of equity-focused implementation theories, models and frameworks in healthcare and their application in addressing ethnicity-related health inequities. Implementation Science 18:51. 10.1186/s13012-023-01304-0

48. Woodward EN, Matthieu MM, Uchendu US, Rogal S, Kirchner JE (2019) The health equity implementation framework: proposal and preliminary study of hepatitis C virus treatment. Implementation Science 14:26. 10.1186/s13012-019-0861-y

49. Stratil JM, Biallas RL, Movsisyan A, Oliver K, Rehfuess EA (2024) Development of an overarching framework for anticipating and assessing adverse and other unintended consequences of public health interventions (CONSEQUENT): a best-fit framework synthesis. BMJ Public Health 2:e000209. <https://doi.org/10.1136/bmjph-2023-000209>

**Tables, figures and info boxes**

**Table 1:** Networks and publication organs with relevance for implementation science

| **Title** | **Description** | **Website** |
| --- | --- | --- |
| *Networks* | | |
| Deutsche Netzwerk Versorgungsforschung e.V. (DNVF), Arbeitsgruppe Implementierungswissenschaft und -praxis in der Versorgungsforschung | Network for scientists involved in improving healthcare and patient care from a scientific, practical or health policy perspective; a working group on implementation science and practice in healthcare research has been existing since 2024 | <https://www.dnvf.de/%C3%BCber-uns/%C3%BCber-das-netzwerk.html> |
| Implementierungs-Netzwerk für Forschung und Praxis (INFo-P) | Implementation network for research and practice for networking in German-speaking countries (including Switzerland and Austria) | <https://www.implementierung.eu/> |
| German Alliance for Global Health Research (GLOHRA) | Focus on global health; brings together scientists interested in implementation science in various formats and also offers funding for research in this context | <https://globalhealth.de/about.html>. |
| European Implementation Collaborative (EIC) | European forum for researchers, practitioners and policy makers interested in the research and practice of health intervention implementation | <https://www.uni-heidelberg.de/de/studium/alle-studienfaecher/versorgungsforschung-und-implementierungswissenschaft-im-gesundheitswesen/versorgungsforschung-und-implementierungswissenschaft-im-gesundheitswesen-master> |
| Global Implementation Society (GIS) | Network focusing on the promotion and establishment of coherent and cooperative approaches for implementation practice, science and politics | <https://globalimplementation.org/> |
| Society for Implementation Research Collaboration (SIRC) | International scientific society dedicated to fostering communication and collaboration between researchers and stakeholders engaged in evaluating the implementation of evidence-based interventions | <https://societyforimplementationresearchcollaboration.org/> |
| *Publication Outlets* | | |
| Implementation Science | Leading international journal in implementation science; focus on research on the implementation of interventions in healthcare | <https://implementationscience.biomedcentral.com/> |
| Implementation Science Communications | Leading international journal in implementation science; focus on research on the implementation of interventions in healthcare | <https://implementationsciencecomms.biomedcentral.com/> |
| Journal Global Implementation Research and Applications | Official journal of the Global Implementation Society; forum for the development, integration and exchange of knowledge and experience in the field of implementation of measures in various areas | <https://link.springer.com/journal/43477> |
| Journal of Implementation Science | International journal; focus on promoting the practice of implementation science | <https://openaccesspub.org/journal/implementation-science> |
| Frontiers in Health Services, Section Implementation Science | International journal; focus on articles that advance the development of implementation science in health and social care | <https://www.frontiersin.org/journals/health-services/sections/implementation-science> |
| Implementation Research and Practice | International journal on interdisciplinary research to implement effective approaches; focus on mental illness and addiction, with an emphasis on vulnerable groups | <https://us.sagepub.com/en-us/nam/implementation-research-and-practice/journal203691> |
| Health Care Research & Implementation (as supplement of the journal “Das Gesundheitswesen”) | German-language journal, focus on studies with practical relevance from care and implementation research | <https://dnvf.de/ver%C3%B6ffentlichungen/dnvf-journal.html> |
| Health Policy & Planning | Journal of health policy and health systems research with a focus on low- and middle-income countries; addresses issues relevant to policy makers, researchers and practitioners; focus on health policy and population-based interventions | <https://academic.oup.com/heapol> |

***Figure 1:*** *Schematic representation of thematic foci and references of implementation research in the field of public health and health services research*


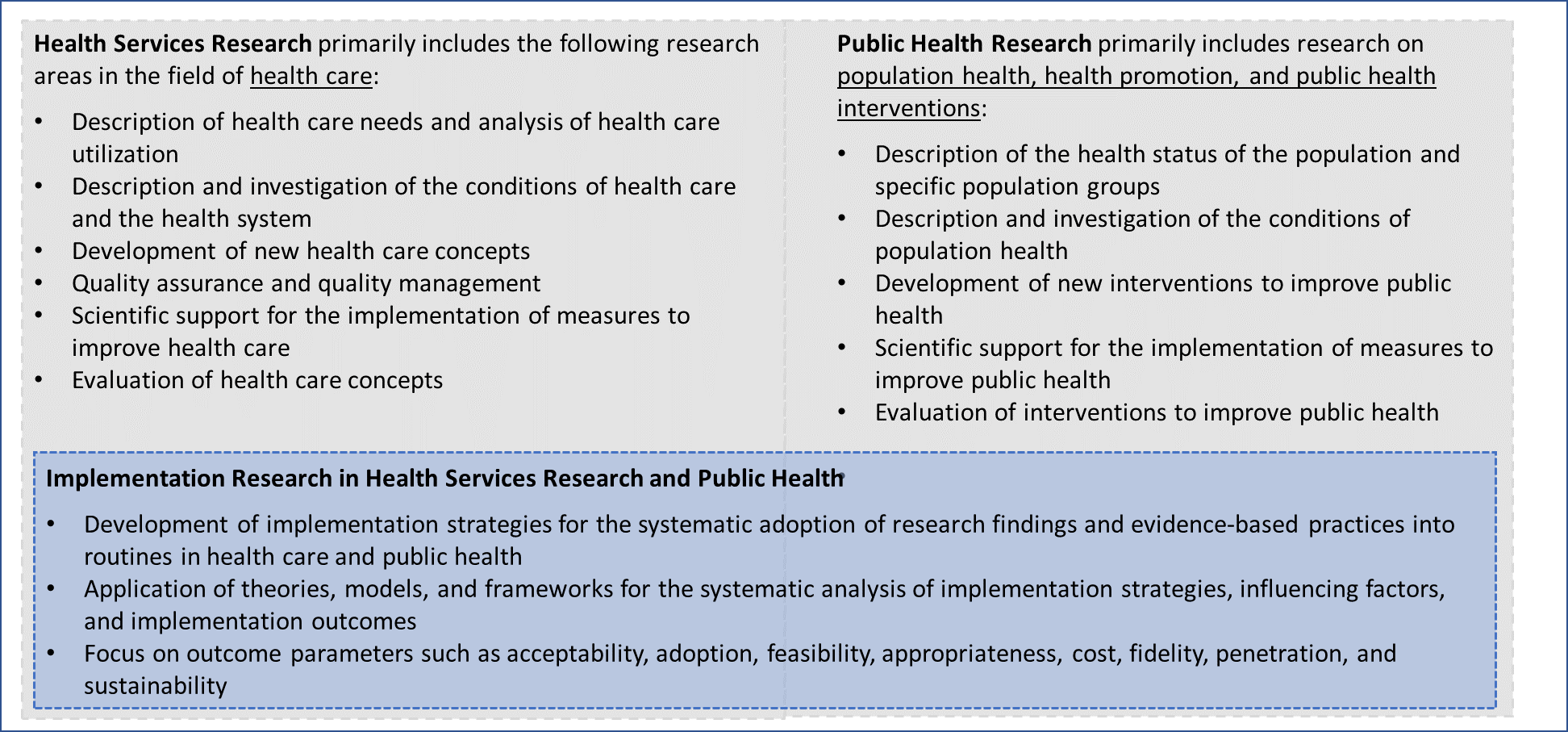


1. <https://www.bewegung-bei-krebs.org/bbk> [accessed 28 January 2025]. [↑](#footnote-ref-1)
2. <https://move-onko.de/> [accessed 28 January 2025]. [↑](#footnote-ref-2)
3. <https://itcproject.org/> [accessed 28 January 2025]. [↑](#footnote-ref-3)
4. <https://www.fruehehilfen.de/forschung-im-nzfh/implementierungsforschung/> [accessed 28 January 2025]. [↑](#footnote-ref-4)
5. <https://www.klinikum.uni-heidelberg.de/personen/prof-dr-michel-wensing-7511> [accessed 28 January 2025]. [↑](#footnote-ref-5)
6. <https://www.uni-bremen.de/institut-fuer-public-health-und-pflegeforschung/abteilungen-arbeitsgruppen/pflegeforschung/pflegewissenschaftliche-evaluations-und-implementierungsforschung>, <https://www.bips-institut.de/en/the-institute/departments/prevention-and-evaluation/implementation-research-and-mental-health.html> [accessed 28 January 2025]. [↑](#footnote-ref-6)
7. <https://www.foga-foerderprogramm.de/DE/GefoerderteVorhaben/Projektsteckbrief-Professur-13FG0003.html> [accessed 28 January 2025]. [↑](#footnote-ref-7)
8. <https://implementation.eu/training-and-education/> [accessed 28 January 2025]. [↑](#footnote-ref-8)
9. <https://www.uni-heidelberg.de/de/studium/alle-studienfaecher/versorgungsforschung-und-implementierungswissenschaft-im-gesundheitswesen/versorgungsforschung-und-implementierungswissenschaft-im-gesundheitswesen-master> [accessed 28 January 2025]. [↑](#footnote-ref-9)
10. <https://www.fliedner-fachhochschule.de/zertifikatsstudium/evidenzbasierte-gesundheitsversorgung-mas/implementierungsmanager-healthcare/> [accessed 2 March 2025]. [↑](#footnote-ref-10)
11. https://www.hs-osnabrueck.de/module/22m0901/

    <https://www.uni-luebeck.de/index.php?id=12828&tx_webparser_pi1%5Bmodulid%5D=2405> [accessed 28 January 2025]. [↑](#footnote-ref-11)
12. <https://implementation.eu/how-is-implementation-science-doing-in-german-speaking-countries/> [accessed 28 January 2025]. [↑](#footnote-ref-12)
13. <https://dnvf.de/ver%C3%B6ffentlichungen/dnvf-journal.htm> [accessed 28 January 2025]. [↑](#footnote-ref-13)
